# Clonal heterogeneity by fluorescence in situ hybridization in multiple myeloma: enhanced cytogenetic risk stratification

**DOI:** 10.1186/s43042-022-00220-0

**Published:** 2022-03-16

**Authors:** Hadeel Yaseen Abdel-Qader, Dina Adel Fouad, Soha Ahmed Abuelela, Heba Mohamed Atif Ismail, Noha Hussein Boshnaq

**Affiliations:** Clinical Pathology Department, Faculty of Medicine, Ain Shams University, Cairo, Egypt

**Keywords:** Multiple myeloma, Chromosomal aberrations, FISH technique, Interleukin 6, Hyperdiploidy

## Abstract

**Background:**

Multiple myeloma (MM) is a proliferation of monoclonal plasma cells that accumulate in bone marrow, leading to bone destruction and marrow failure. Cytogenetic analysis is a challenge in MM because of the low mitotic activity and the rapid loss of plasma cells viability in bone marrow culture. Adding mitogens such as interleukin 6 (IL6) is known to promote the in vitro growth of myeloma cell lines and enhance the fluorescence in situ hybridization application. This study aims to evaluate the prognostic impact of cytogenetic abnormalities detected by enhanced interphase fluorescence in situ hybridization (iFISH) technique in Egyptian MM patients.

**Results:**

Patients who had hyperdiploidy significantly presented with higher Hb level and lower calcium levels compared to non-hyperdiploid patients. They were staged as stage I and II by International staging system (ISS) and considered as standard risk showing better response to treatment. On the contrary, features associated with a worse outcome were patients having del 17p and those belonged to intermediate and high risk groups.

**Conclusion:**

In conclusion, adding interleukin 6 to MM cell culture promotes the in vitro growth of myeloma cells and enhances the successful application of FISH technique. A comprehensive FISH probe set investigating high, intermediate and low-risk cytogenetic abnormalities is needed for accurate risk stratification. Hyperdiploid-myeloma is a favorable risk genetic subtype of MM associated with rapid response to therapy compared to patients having del 17p, t(4;14), and other 14q rearrangements rather than t(11;14) and t(6;14).

## Background

Multiple myeloma (MM) accounts for approximately 1% of all malignant diseases, 10% of all hematological malignancies in whites and 20% in African Americans [1]. Cytogenetic abnormalities have emerged as the most important prognostic factor in MM and include interphase fluorescence in situ hybridization (iFISH) with enrichment of CD138+ cells especially in samples having less plasma cells as the standard test [2]. However, cytogenetic analysis remains a challenge in myeloma patients because of the low proliferation of malignant plasma cells [3].

More than half of MM cases have a hyperdiploid karyotype, characterized by trisomies involving odd chromosomes which are associated with a favorable outcome. Hypodiploid karyotype can be found in about 40% of patients. Three recurrent genetic abnormalities, t(4;14), deletion(17p) and t(14;16), are mostly associated with a poorer outcome. Chromosome 1 abnormalities are also adverse prognostic factors [4]. The risk stratification of newly diagnosed MM includes deletion of chromosome 17p, t(14;16), and t(14;20) as high-risk group, t(4;14), del 13q and hypodiploidy as intermediate-risk, t(11;14), t(6;14), and hyperdiploidy as low-risk group [5].

The prognosis of myeloma was determined earlier by International staging system (ISS) based on beta-2 Microglobulin and Albumin. It has been demonstrated that combining FISH and serum lactate dehydrogenase (LDH), along with the ISS stage in this new and revised ISS (R-ISS), could significantly improve the prognostic assessment in terms of progression-free survival (PFS) and overall survival (OS), eventually leading to enhancement of the efficacy of the therapeutic approaches [6]. Our study aims to evaluate the prognostic impact of chromosomal abnormalities through an expanded enhanced FISH panel in newly diagnosed Egyptian MM patients, in relation to the well-established prognostic factors and to determine their clinico-pathological significance.

## Methods

### Patients and methods

Our study was conducted on 60 newly diagnosed Egyptian patients diagnosed as multiple myeloma according to International Myeloma Working Group criteria [7], 37 (61.7%) were males and 23 (38.3%) were females, aged between 39 and 81 years, who were enrolled after obtaining an informed written consent and a detailed case history. The study was conducted at the Department of Clinical Pathology, Ain Shams University Hospitals, and was approved by the local ethical committee of Ain Shams University. Each participant provided written consent.

Heparinized bone marrow samples (2–3 mL) were analyzed using karyotyping and enhanced iFISH by (Interleukin 6) to promote the in vitro growth of myeloma cell lines and enhance the FISH application [8]. Lyophilized and stabilized human IL6 (Origene, Finland) reconstituted with sample diluent was added to the tissue culture media (MarrowMax, Gibco, USA) and left for 72 h.

Patients’ samples were assessed using the following set of FISH probes, locus specific identifier (LSI) D13S319 (13q14.3) probe, LSI IGH dual color break apart rearrangement probe (14q32), LSI IGH/CCND1 dual color dual fusion probe, LSI 17p.13/CEP 17 probe, LSI D5S23, D5S721/CEP9/CEP15 FISH Probe and LSI IGH/FGFR 3 DF FISH probe.

Determination of the cutoff levels of sets was performed by counting 200 nuclei in 5 negative controls (peripheral blood lymphocytes from healthy donors) for each probe set using Olympus BX-51 microscope (Olympus, Tokyo, Japan). The images were captured and analyzed with the CytoVision Capture A4 software (Leica Microsystems, USA). The cutoff level for all the probes was 10% [9].

### Statistical analysis

Data were collected, revised, coded and entered to the Statistical Package for Social Science (IBM SPSS) version 23. The quantitative data were presented as mean, standard deviations and ranges for parametric data and median, inter-quartile range (IQR) for nonparametric sets. The comparisons of qualitative data were done by using Chi-square test and Fisher exact test instead, only when the expected count in any cell found less than 5. Univariate and multivariate regression analysis was used to assess predictors of response. The confidence interval was set to 95%, and the margin of error accepted was set to 5%. So, the *P* value was considered significant when *P* > 0.05.

## Results

Cytogenetics abnormalities were detected in 45 patients (75%), and they were distributed as follows: hyperdiploidy in 35.0%, 17p deletion representing 11.7%, t(4;14) representing 10.0%, del 13q representing 6.7%, t(11;14) representing 6.7% and 14q deletion representing 3.3% of all cases and t(6;14) 1.7% of cases.

Most of our patients who had hyperdiploidy significantly presented with elevated Hb level, low calcium levels, were staged as stage I and II ISS and considered as standard risk group, therefore showed better response to treatment Table 1 and those with del 13 q, their clonal plasma cells showed significant lambda chain restriction (*P* value < 0.05), while patients who harbored 17 p deletion were significantly presented with hypercalcemia and considered as stage III ISS and RISS; hence, they showed poor response to treatment as shown in Table 1. Regarding 14q rearrangement in MM, all t(4;14) positive patients were significantly staged as stage III RISS, while all patients who harbored t (6;14) and t (11;14) were significantly staged as stage II RISS. On the other hand, comparing t(11;14) with the studied parameters did not attain any statistically significant association (*P* value > 0.05).

**Table 1:** Comparison of cytogenetic aberrations and the studied clinical parameters in our patients

	Hyperdiploidy		*P* value	t(4;14)		*P* value	del 17 p		*P* value
	Negative	Positive		Negative	Positive		Negative	Positive	
	No. = 39	No. = 21		No. = 54	No. = 6		No. = 53	No. = 7	
Hb (gm/dL)									
≥ 10	9 (23.1%)	11 (52.4%)	**0.022**	20 (37.0%)	0 (0.0%)	0.068	20 (37.7%)	0 (0.0%)	**0.047**
< 10	30 (76.9%)	10 (47.6%)		34 (63.0%)	60 (100.0%)		33 (62.3%)	7 (100.0%)	
Creat (mg/dL)									
≤ 2	21 (53.8%)	16 (76.2%)	0.090	35 (64.8%)	2 (33.3%)	0.132	35 (66.0%)	2 (28.0%)	0.055
> 2	18 (46.2%)	5 (23.8%)		19 (35.2%)	4 (66.7%)		18 (34.0%)	5 (71.4%)	
Ca (mg/dL)									
≤ 11	25 (64.1%)	12 (57.1%)	0.597	35 (64.8%)	2 (33.3%)	0.132	37 (69.8%)	0 (0.0%)	**0.00**
> 11	14 (35.9%)	9 (42.9%)		19 (35.2%)	4 (66.7%)		16 (30.2%)	7 (100.0%)	
BJP									
Negative	11 (28.2%)	6 (28.6%)	0.976	15 (27.8%)	2 (33.3%)	0.774	0 (0.0%)	2 (28.6%)	0.988
Positive	28 (71.8%)	15 (71.4%)		39 (72.2%)	4 (66.7%)		53 (100.0%)	5 (71.4%)	
IPT									
Lambda	19 (48.7%)	5 (23.8%)	0.060	21 (38.9%)	3 (50.0%)	0.598	17 (32.1%)	7 (100.0%)	**0.001**
Kappa	20 (51.3%)	16 (76.2%)		33 (61.1%)	3 (50.0%)		36 (67.9%)	0 (0.0%)	
Response to treatment									
Non-responder	19 (48.7%)	2 (9.5%)	**0.002**	18 (33.3%)	3 (50.0%)	0.417	14 (26.4%)	7 (100.0%)	**0.00**
Responder	20 (51.3%)	19 (90.5%)		36 (66.7%)	3 (50.0%)		39 (73.6%)	0 (0.0%)	
ISS stage									
I	15 (38.5%)	10 (47.6%)	**0.027**	23 (42.6%)	2 (33.3%)	0.083	25 (47.2%)	0 (0.0%)	**0.00**
II	8 (20.5%)	9 (42.9%)		17 (31.5%)	0 (0.0%)		17 (32.1%)	0 (0.0%)	
III	16 (41.0%)	2 (9.5%)		14 (25.9)	4 (66.7%)		11 (20.8%)	7 (100.0%)	
RISS stage									
I	4 (10.3%)	3 (14.3%)	**0.007**	7 (13.0%)	0 (0.0%)	**0.000**	7 (13.2%)	0 (0.0%)	**0.00**
II	21 (53.8%)	18 (85.7%)		39 (72.2%)	0 (0.0%)		39 (73.6%)	0 (0.0%)	
III	14 (35.9%)	0 (0.0%)		8 (14.8%)	6 (100.0%)		7 (13.2%)	7 (100.0%)	

* values at risk

*ISS* international staging system, *RISS* revised international staging system, *HB* hemoglobin, *Creat* serum creatinine, *Ca* serum calcium, *IPT* immunophenotyping

*Chi-square test

*P* value > 0.05: non-significant; *P* value < 0.05: significant (bold); *P* value < 0.01: highly significant

Regarding risk stratification, most of our standard risk patients significantly had low calcium level, showed kappa light chain restriction and were mostly staged as stage I and II ISS and RISS, while most of our high risk patients significantly had anemia, hypercalcemia, had lambda restriction and were staged as stage III ISS and RISS as shown in Table 2.

**Table 2:** Association between risk stratification and response to treatment with the studied parameters

	Risk stratification			*P* value	Response to treatment		*P* value
	Standard risk	Intermediate risk	High risk		Non-responders	Responders	
	No. = 41	No. = 10	No. = 9		No. = 21	No. = 39	
Sex							
Females	16 (39.0%)	4 (40.0%)	3 (33.3%)	0.944	10 (47.6%)	13 (33.3%)	0.278
Males	25 (61.0%)	6 (60.0%)	6 (66.7%)		11 (52.4%)	26 (66.7%)	
HB (gm/dL)							
≥ 10	20 (48.8%)	0 (0.0%)	0 (0.0%)	**0.001**	1 (4.8%)	19 (48.7%)	**0.001**
< 10	21 (51.2%)	10 (100.0%)	9 (100.0%)		20 (95.2%)	20 (51.3%)	
Creat (mg/dL)							
≤ 2	29 (70.7%)	4 (40.0%)	4 (44.4%)	0.103	11 (52.4%)	26 (66.7%)	0.278
> 2	12 (29.3%)	6 (60.0%)	5 (55.6%)		10 (47.6%)	13 (33.3%)	
Ca (mg/dL)							
≤ 11	32 (78.0%)	4 (40.0%)	1 (11.1%)	**0.000**	9 (42.9%)	28 (71.8%)	**0.028**
> 11	9 (22.0%)	6 (60.0%)	8 (88.9%)		12 (57.1%)	11 (28.2%)	
Positive	41 (100.0%)	10 (100.0%)	9 (100.0%)				
IPT							
Lambda	9 (22.0%)	7 (70.0%)	8 (88.9%)	**0.000**	16 (76.2%)	8 (20.5%)	**0.000**
Kappa	32 (78.0%)	3 (30.0%)	1 (11.1%)		5 (23.8%)	31 (79.5%)	
ISS							
I	21 (51.2%)	2 (20.0%)	2 (22.2%)	**0.000**	3 (14.3%)	22 (56.4%)	**0.000**
II	15 (36.6%)	2 (20.0%)	0 (0.0%)		2 (9.5%)	15 (38.5%)	
III	5 (12.2%)	6 (60.0%)	7 (77.8%)		16 (76.2%)	2 (5.1%)	
RISS							
I	7 (17.1%)	0 (0.0%)	0 (0.0%)	**0.000**	3 (14.3%)	4 (10.3%)	**0.003**
II	34 (82.9%)	3 (30.0%)	2 (22.2%)		8 (38.1%)	31 (79.5%)	
III	0 (0.0%)	7 (70.0%)	7 (77.8%)		10 (47.6%)	4 (10.3%)	

* values at risk

*ISS* international staging system, *RISS* revised international staging system, *HB* hemoglobin, *Creat* serum creatinine, *Ca* serum calcium, *IPT* immunophenotyping

*Chi-square test

*P* value > 0.05: non-significant; *P* value < 0.05 (bold): significant (bold); *P* value < 0.01: highly significant

As for treatment response, non-responders to treatment were found to significantly have hypercalcemia, anemia, Lambda restriction and were staged as stage III according to ISS and RISS as shown in Table 2.

**Fig. 1:**
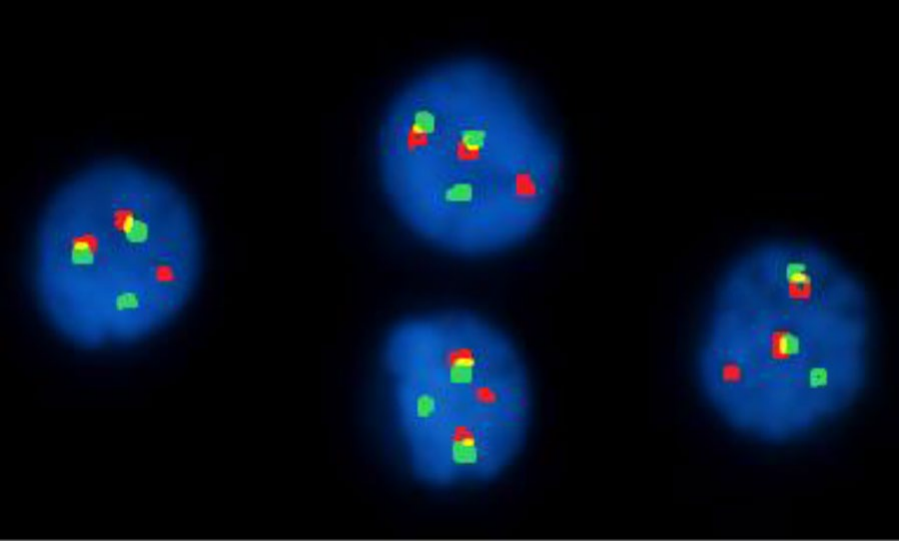
Interphase FISH analysis using LSI IGH/FGFR3 DF probe showing t(4′14)(p16;q32)(FGFR3/IGH). IGH in green and FGFR3 in red

**Fig. 2:**
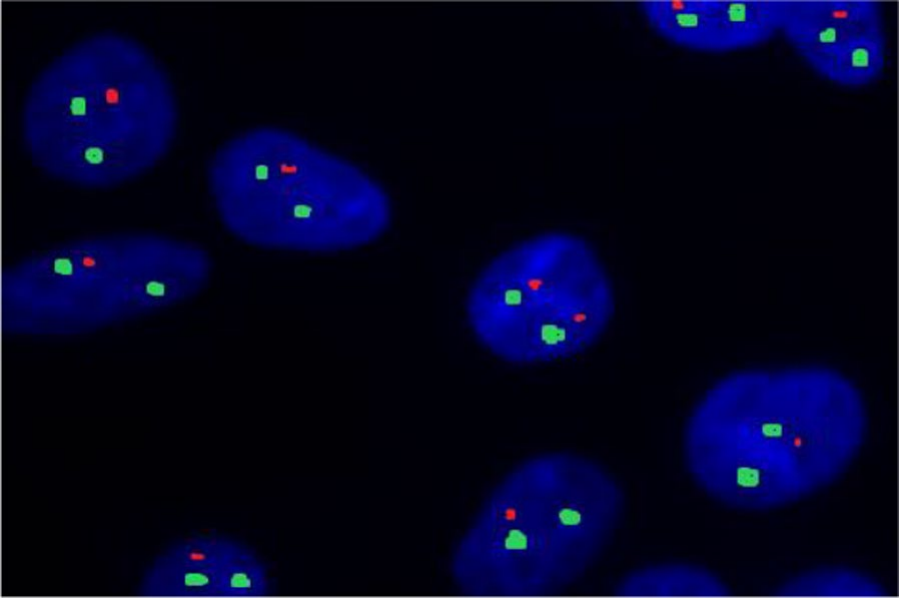
Interphase FISH analysis using Vysis LSI TP53/CEP 17 FISH Probe showing positive result for heterozygous deletion of TP53(17p13). TP53(17p13) in red and CEP 17 in green

**Fig. 3:**
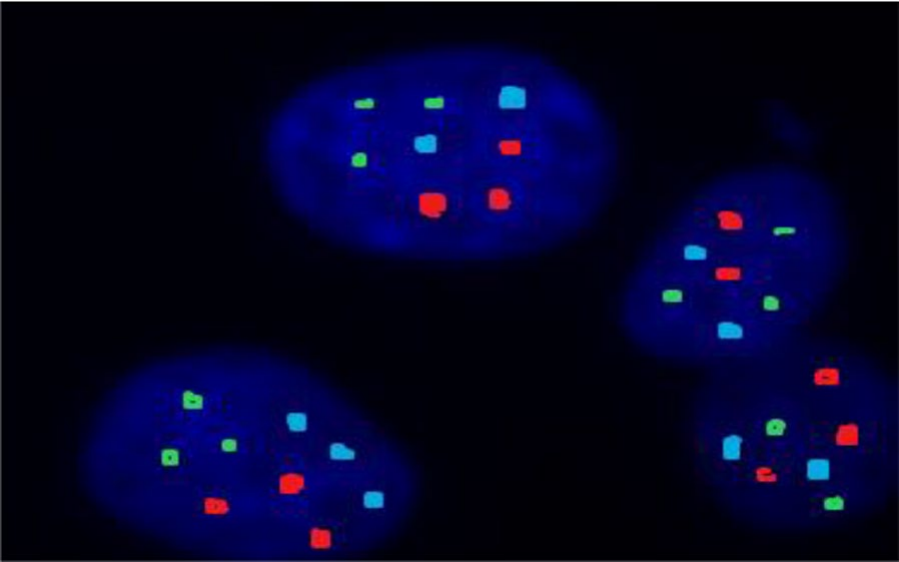
Interphase FISH using Vysis LSI D5S23, D5S721/CEP 9/CEP 15 FISH Probe showing abnormal cells containing hyperdiploidy of chromosome 5 (three green signals), and chromosome 15 (three red signals)

**Fig. 4:**
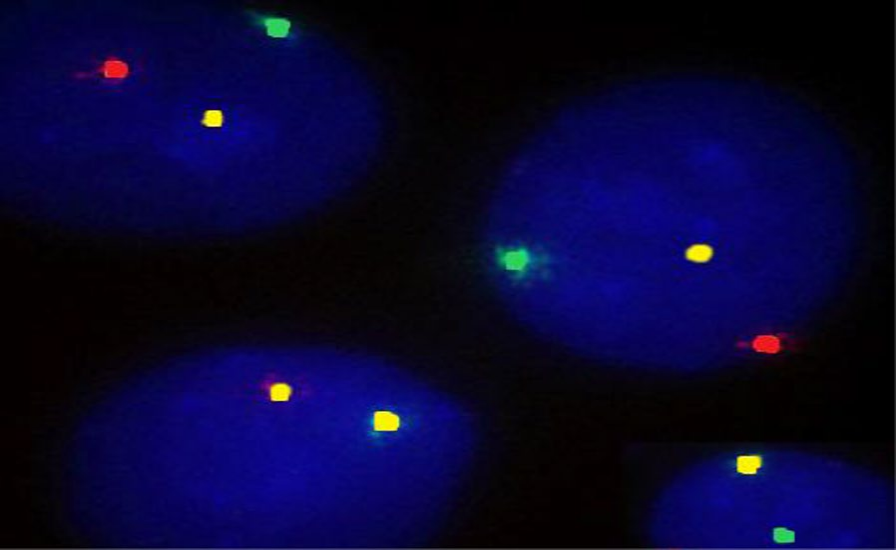
Interphase FISH analysis using Vysis LSI IGH dual color break apart rearrangement probe showing positive result for IGH rearrangements

Standard risk cytogenetic aberrations represented 68.3% in the form of t(6;14) and t(11;14), hyperdiploidy and normal karyotype with absence of cytogenetic aberrations by FISH. Intermediate risk represented 16.7% of patients. Meanwhile, high risk aberrations in the form of del 17p and other 14 q rearrangements represented 15% of our studied patients. The major features associated with good treatment response were hyperdiploidy and patients who belonged to the standard risk group. On the contrary, features associated with a worse outcome were patients harboring del17p and those belonged to intermediate and high risk groups (Table 3; Figs. 1, 2, 3, 4).

**Table 3:** The association between treatment response and risk stratification with cytogenetic aberrations

	Non-responders	Responders	Test value	*P* value	Sig.
	No. = 21	No. = 39			
FISH					
Negative	4 (19.0%)	11 (28.2%)	0.611	0.434	NS
Hyperdiploidy	2 (9.5%)	19 (48.7%)	9.217	0.002	**HS**
t(4;14)	3 (14.3%)	3 (7.7%)	0.659	0.417	NS
del13q	3 (14.3%)	1 (2.6%)	3.014	0.083	NS
t(11;14)	2 (9.5%)	2 (5.1%)	0.424	0.515	NS
del17p	7 (33.3%)	0 (0.0%)	14.717	0.000	**HS**
14q	0 (0.0%)	3 (7.7%)	1.700	0.192	NS
Risk stratification					
Standard risk	8 (38.1%)	33 (84.6%)	14.310*	0.001	**HS**
Intermediate risk	6 (28.6%)	4 (10.3%)			
High risk	7 (33.3%)	2 (5.1%)			

*FISH* fluorescence in situ hybridization

*Chi-square test

*P* value > 0.05: non-significant; *P* value < 0.05: significant (bold); *P* value < 0.01: highly significant

Multivariate logistic regression analysis showed that kappa light chain restriction and staging by both ISS and RISS together with hyperdiploidy are the most important prognostic factors of treatment response as shown in Table 4.

**Table 4:** Multivariate and univariate logistic regression analysis for predictors of treatment response

	Univariate logistic regression analysis		Univariate logistic regression analysis	
	*B*	*P* value	*B*	*P* value
IPT (Kappa)	4.083	**0.003**	2.518	**0.00**
ISS	− 3.937	**0.001**	− 2.125	**0.00**
RISS	3.597	**0.015**	− 1.067	**0.040**
Hyperdiploidy	3.331	**0.031**	2.200	**0.007**

*P* value < 0.05: significant (bold)

*IPT* immunophenotyping, *ISS* international staging system, *RISS* revised international staging system

**Fig. 5:**
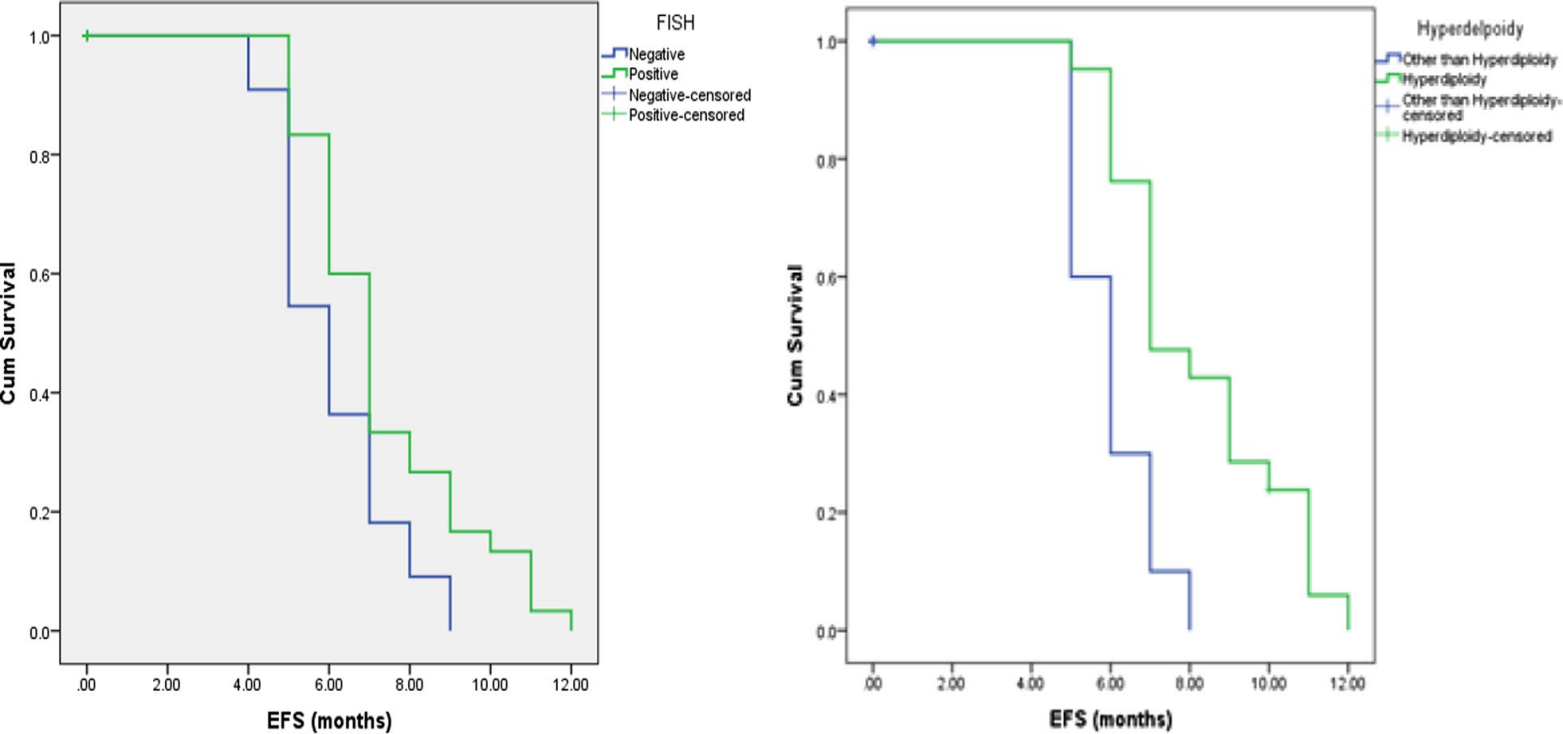
Kaplan–Meier analysis for the relation between FISH results (left) and hyperdiploidy (right) and event free survival among the studied patients

Follow-up and Kaplan–Meier survival analysis revealed patients with hyperdiploidy showed a longer event free survival. However, neither 14q rearrangement nor risk stratification affected event free survival among our studied patients as shown in Table 5 and Fig. 5.

**Table 5:** Kaplan–Meier analysis for the relation between FISH results and hyperdiploidy with event free survival among the studied patients

	Mean	SE	95% CI		Median	SE	95% CI		Log Rank test		
			Lower	Upper			Lower	Upper	*X*2	*P* value	Sig.
FISH											
Negative	6.091	0.456	5.196	6.985	6	0.532	4.958	7.042	3.949	0.047	**S**
Positive	7.367	0.37	6.642	8.091	7	0.323	6.367	7.633			
Hyperdiploidy											
Other than hyperdiploidy	6.000	0.333	5.347	6.653	6.000	0.483	5.053	6.347	10.548	0.001	**HS**
Hyperdiploidy	8.202	0.466	7.288	9.116	7.000	0.654	5.718	8.282			

*P* value < 0.05: significant (bold)

*FISH* fluorescence in situ hybridization

## Discussion

Multiple myeloma (MM) is a disease characterized by heterogeneity in clinical presentations, genetic abnormalities, and in clinical outcome. Cytogenetic abnormalities in MM have emerged as the most important factor that determine the prognosis and survival. Although the conventional karyotyping offers a full view of chromosomes, yet its application is limited by the low mitotic activity of myeloma cells. On the other hand, fluorescence in situ hybridization (FISH) can detect a greater number of abnormalities and hence has become the standard test in determining genetic abnormalities in MM [2].

Out of the 60 patients included in our study, with male to female ratio of 1.6:1. Their age ranged from 39 to 81 years old with a mean of 59 years which was in agreement with both Khan et al., 2017 and Udupa et al., 2020 [2, 10]. The most common chromosomal aberrations in newly diagnosed MM are hyperdiploidy and in the second place 13q del, while least common abnormalities are t(14;20) and t(6;14) [3]. Trisomies of odd numbered chromosomes are seen in nearly half of patients with MM and typically correlate with a hyperdiploid state and better overall survival (OS) [11].

Karyotype and FISH analysis revealed that hyperdiploidy is the most common chromosomal aberration found in 35% of patients. Al Hashmi and his colleagues, in 2019, study showed that 50% of their patients had hyperdiploidy which is higher than the ratio in our study [12]. Also in line with our study, hyperdiploidy was the most common cytogenetic aberration in a study by White et al., 2018 [13]. On the contrary in a study in 2020 by Hamdaoui et al., the Moroccan population gains of chromosomes 3, 5, 9, 15, and 19 represented (9%) of cases; however, this may be because their results depended on karyotype result [14].

In the present study, hypercalcemia (Ca > 11 mg/dL) was found in 38.3% of patients and a similar ratio was found in the study by Khallaf et al., 2020 [15]. Median Serum creatinine of 1.7 mg/dL was detected in the present study which was about the same result 1.7 ± 2.1 detected by Udupa et al., 2020 [2]. Renal impairment (serum creatinine > 2 mg/dL) in our study was detected in 38.3% of patients but a lower ratio was detected in 2020 by both Khallaf et al. and Warnnissorn et al. [15, 16]. The higher ratio of renal impairment in this study may be attributed to the higher ratio of renal failure among Africans as stated by an African study in 2018 by Cisse et al. [17].

Regarding prognostic parameters included in our study, β2 microglobulin had a median of 3.5 mg/L which is lower than the result detected by Udupa et al., 2020 while serum albumin showed a mean ± SD of 3.4 ± 0.6. This result was slightly lower than that of Udupa et al., 2020 which was in g/dL 3.5. Lactate dehydrogenase enzyme (LDH) in our study showed a mean ± SD of 357.7 ± 114.8 which was slightly lower in Udupa et al., 2020 being 254 ±112.4 [2].

Concerning risk stratification, our study shows that 41/60 patients as standard risk representing (68.3%), 10/60 patients showed intermediate risk (16.7%) and 9/60 high risk (15.0%). A distribution was made in 2015 by Zeng et al. who retrospectively analyzed 67 consecutive myeloma patients with 26 low-risk patients (38.8%), 24 intermediate-risk (35%) and totally 17 high-risk (25%) were enrolled; this discrepancy between our studies may be because their study was based on both fluorescence in situ hybridization and ISS stage [18].

In the present study, staging by ISS and RISS were found to be highly statistically significant with risk stratification. However, in a study by Amere et al. (2016), there was no correlation between high risk cytogenetics and ISS-III [19]. In Udupa et al. 2020, there was a 66.4% moderate correlation between ISS-III and high-risk cytogenetics which was statistically insignificant with *P* value of 0.213 [2].

By applying multivariate logistic regression analysis, the present study showed that the most important predictors of the response to treatment was found to be kappa light chain restriction and staging by both ISS and RISS together with hyperdiploidy with *P* values (0.003, 0.001, 0.015 and 0.031 respectively). Similarly in a recent study on 292 newly diagnosed MM patients by Barilà et al., 2020, concomitant trisomies 9/11/15 were the most powerful prognostic factor (*P* < 0.01), followed by IGH rearrangement (*P* < 0.04), although in the opposite direction [20]. Also Mellors et al. in 2020 revealed that R-ISS stage and age 70 years were associated with inferior OS in the base model [21].

The present study found that 39/60 (65%) were responders to treatment while 21/60 (35.0%) were non-responders. Non-responders were found to significantly have hypercalcemia, anemia, Lambda restriction and were staged as stage III according to ISS and RISS. Interestingly, Tandon et al., 2019 observed that patients who demonstrated high-risk features, such as renal impairment and high-risk cytogenetics [specifically t(4;14)], as well as those with ISS stage III disease, were more likely to achieve a transient rapid response [22]. It is possible that such patients have a higher plasma cell proliferative rate, resulting in increased initial sensitivity to treatment, but a higher rate of loss of response, resulting in inferior long-term outcomes that have been well described in these patients.

Hyperdiploidy in our study was highly significantly associated with the response to treatment (where 90% of hyperdiploidy were responders), and with staging using (RISS) (where 14.3% of hyperdiploidy patients were stage I, 85.7% were stage II while no patients were stage III), also it was significantly associated with the hemoglobin level and with staging using (ISS). In line with our study, a recent Spanish study by Benavides et al., 2021 has shown that hyperdiploid patients have better response rates to treatment and longer survival than patients with other aberrations [23]. Similarly Barilà and his colleagues in 2020 stated that features associated with a better outcome were co-occurrence of trisomy of 9/11/15 chromosomes and autologous stem cell transplantation (ASCT) [20].

Kaplan–Meier analysis showed that hyperdiploidy was associated with higher overall survival. Similarly in 2019, Al Hashmi et al. addressed the question of whether hyperdiploidy can affect survival in the presence of concomitant high/intermediate risk cytogenetics [12]. Data from the high + intermediate risk category grouped into hyperdiploid and non-hyperdiploid were analyzed by Kaplan–Meier survival analysis. In both progression-free and overall survival analyses, they observed > 50% median survival in hyperdiploid cases compared to non-hyperdiploid cases. Progression-free survival was found to be significantly higher in the hyperdiploidy (HD) group compared to the non-hyperdiploidy (N) group, indicating that the risk of death in the non-hyperdiploid group was twice that of the hyperdiploid group.

The second most common cytogenetic aberration in our study was 17p deletion representing (11.7%) of all cases. All studied MM patients having 17p deletion were significantly presented with hypercalcemia and lambda restriction. They were also significantly defined as ISS and RISS stage III as they were non-responders to treatment. Similarly Jakubowiak et al., 2013 detected that del 17p13 assessed by FISH is usually a late event in the evolution of MM [24]. It is indicative of more aggressive disease and has consistently been shown to be one of the more significant prognostic factors among high-risk cytogenetic abnormalities regardless of the treatment strategy, including conventional chemotherapy or targeted therapies. Further studies by Lakshman et al., 2019 show that patients with acquisition of del(17p) on follow-up are associated with marked reduction in OS when compared with patients who do not acquire del(17p) and provide estimates for expected outcomes in patients who acquire del(17p) [25]. However, our analysis did not allow for a complete assessment of all potential high risk abnormalities including the duplication 1q and deletion 1p due to some funding issues, and future studies are recommended to take into account these emerging factors in order to fully understand the individual contributions of each abnormality.

## Conclusion

In conclusion, hyperdiploid-myeloma is a favorable risk genetic subtype of MM associated with favorable outcome and rapid response to therapy. It showed a significant prognostic impact in relation to the well-established prognostic factors. Monitoring the patients' response to treatment confirmed its association with good response. Including the hyperdiploid FISH panel of probes in routine FISH analysis of myeloma patient is crucial for accurate risk stratification.

## Data Availability

The datasets used and analyzed in this study are available from the corresponding author upon reasonable request.

## Abbreviations

β2M: β2-Microglobulin
CD: Cluster of differentiation
CDK: Cyclin-dependent kinase
CEP: Chromosome enumeration probe
CGH: Comparative genomic hybridization
CR: Complete response
CRAB: Hypercalcemia, renal failure, anemia, bone disease
CRP: C-reactive protein
CT: Computed tomography
DAPI: Diamidino phenylindole dihydrochloride
del: Deletion
DNA: Deoxyribonucleic acid
EDTA: Ethylenediamine-tetraacetic acid
FISH: Fluorescence in situ hybridization
FLC: Free light chain
I-FISH: Interphase fluorescent in situ hybridization
IL: Interleukin
IMWG: The International Myeloma Working Group
IPT: Immunophenotyping
ISS: International staging system
LDH: Lactate dehydrogenase
LSI: Locus specific identifier
M protein: Monoclonal protein
MDEs: Myeloma defining events
OS: Overall survival
p: Short arm of chromosome
PFS: Progression-free survival
q: Long arm of chromosome
RISS: Revised international staging system.
RPMI: Roswell Park Memorial Institute medium
SD: Standard deviation
t: Translocation
TP53: Tumor protein p53
WHO: World Health Organization
